# CONE: A Connected Dominating Set-Based Flooding Protocol for Wireless Sensor Networks

**DOI:** 10.3390/s19102378

**Published:** 2019-05-23

**Authors:** Dennis Lisiecki, Peilin Zhang, Oliver Theel

**Affiliations:** Department of Computer Science, Carl von Ossietzky University of Oldenburg, 26111 Oldenburg, Germany; dennis.lisiecki@uni-oldenburg.de (D.L.); theel@informatik.uni-oldenburg.de (O.T.)

**Keywords:** wireless sensor networks, energy efficiency, flooding, broadcast storm, connected dominating set

## Abstract

Wireless sensor networks (WSNs) play a significant role in a large number of applications, e.g., healthcare and industry. A WSN typically consists of a large number of sensor nodes which rely on limited power sources in many applications. Therefore, improving the energy efficiency of WSNs becomes a crucial topic in the research community. As a fundamental service in WSNs, network flooding offers the advantages that information can be distributed fast and reliably throughout an entire network. However, network flooding suffers from low energy efficiency due to the large number of redundant transmissions in the network. In this work, we exploit connected dominating sets (CDS) to enhance the energy efficiency of network flooding by reducing the number of transmissions. For this purpose, we propose a connected dominating set-based flooding protocol (CONE). CONE inhibits nodes that are not in the CDS from rebroadcasting packets during the flooding process. Furthermore, we evaluate the performance of CONE in both simulations and a real-world testbed, and then we compare CONE to a baseline protocol. Experimental results show that CONE improves the end-to-end reliability and reduces the duty cycle of network flooding in the simulations. Additionally, CONE reduces the average energy consumption in the FlockLab testbed by 15%.

## 1. Introduction

Over the past decade, Wireless Sensor Networks (WSNs) have begun to play a significant role as an enabling technology in a large number of applications, e.g., healthcare, industry, and agriculture, to name a few. A WSN typically contains a large number of sensor nodes. Generally, a sensor node consists of a processing unit, a radio for wireless communication and one or more sensors. Sensor nodes are usually used to monitor environmental conditions, especially in places where wiring is impractical. Presently, WSNs act as an important infrastructure in the popular Internet of Things (IoT). The idea behind IoT is to fully integrate tiny devices such as sensor nodes with IP-based networks, in order to realize new areas of WSN applications [1]. The integration of WSNs in IoT enables users’ immediate access to (environmental) data collected by the sensor nodes. This effectively increases the efficiency and the productivity of many processes [2]. However, in many applications, sensor nodes are powered by a limited power source, and sometimes it is unfeasible to replace or recharge sensor nodes. Hence, the network lifetime becomes a critical concern in the design of WSNs [3]. Increasing the energy efficiency of WSNs is an important topic in the research community [4].

As a fundamental service in WSNs, network flooding offers the advantages that information can be distributed fast and reliably throughout an entire network [5]. It is exploited in data dissemination, remote code update, wireless bulk data transfer, periodical update (e.g., in the IPv6 Routing Protocol for Low-Power and Lossy Networks (RPL) [6]), and so forth. In network flooding, a node called “initiator” typically triggers the flooding process by broadcasting a packet to its neighboring nodes. If a node receives this packet, then it rebroadcasts this packet. Nodes repeat this process until a predefined event occurs, e.g., a timer expires. This process literally “floods” the information to all the nodes in the whole network. Besides, network flooding is simple to implement, and it normally does not require any routing mechanism that maintains routes from sources to certain destinations in a network. Therefore, it is often used to discover neighbors and to construct routing tables during network initialization, e.g., in [7,8].

**Figure 1:**
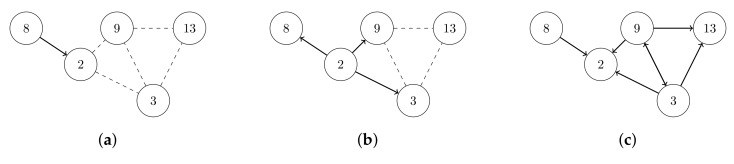
The broadcast storm problem in network flooding. The dashed lines indicate that two nodes are within transmission range of each other. The arrows show a transmission from one node to another one. (**a**) Node 8 broadcasts a packet to node 2. (**b**) Node 2 broadcasts this packet to node 3, 8, and 9. (**c**) Node 8, 9, and 3 broadcast the packet simultaneously to node 2 and others in response.

However, network flooding suffers from the broadcast storm problem [9], leading to low energy efficiency of the network. A broadcast storm occurs when a network is overwhelmed by continuous broadcast traffic. For instance, when different nodes are broadcasting data over a network link and the other nodes are rebroadcasting the data back to the network link in response, then this eventually leads to a network communication breakdown [9]. Correspondingly, the broadcast storm causes collisions, traffic overhead, waste of bandwidth, and so forth. Therefore, it results in extremely energy-inefficient network flooding. As Figure 1 shows, node 2 and its neighboring nodes suffer from infinite broadcasting traffic.

**Figure 2:**
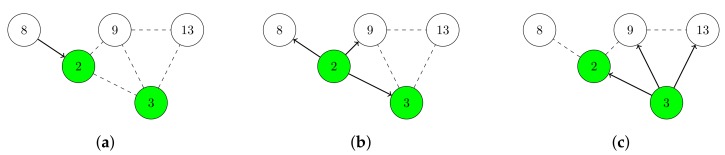
Using a CDS for network flooding. Nodes which are in the CDS are colored in green. The dashed lines indicate that two nodes are in transmission range of each other. Arrows show a transmission from one node to another one. (**a**) Node 8 broadcasts a packet to node 2. (**b**) Since node 2 is in the CDS, it rebroadcasts the packet to node 3, 8, and 9. (**c**) Since node 3 is in the CDS, it rebroadcasts the packet to node 2, 9, and 13. However, because node 8 and 9 are not in the CDS, they do not rebroadcast the packet.

Energy efficiency of network flooding can be enhanced by using a minimal connected dominating set (MCDS). A Connected Dominating Set (CDS) is a connected subgraph of an initial graph that represents a network. Each node that is not in the CDS is adjacent to at least one node of the CDS. A MCDS is a CDS with the minimal number of nodes. MCDS-based approaches reduce communication overhead, radio duty cycles, and overall energy consumption of WSNs [10]. In Figure 2, node 2 and 3 form a CDS of the network. Instead of letting all nodes rebroadcast information, only node 2 and 3 rebroadcast this information. Nevertheless, every node which is not in the CDS also eventually receives the information.

However, finding a MCDS is a Non-deterministic Polynomial-time (NP)-hard problem [8]. There has been a lot of work done on finding approximation algorithms to solve this NP-hard problem [11]. In this article, we propose the Connected Dominating Set-based flooding protocol (called “CONE”), to improve the energy efficiency of network flooding. CONE exploits an approximation algorithm to construct a CDS and subsequently uses this CDS to boost the flooding procedure. As a side effect, CONE significantly reduces the problem of broadcast storms.

For the evaluation of CONE, we perform simulations for analyzing the behavior of CONE. Furthermore, we employ a real-world testbed FlockLab [12] for the evaluation. The evaluation concentrates on (1) packet loss because of IEEE 802.15.4 medium access control (MAC) contention, (2) radio duty cycle (RDC), and (3) energy consumption. These metrics very well indicate the energy efficiency and reliability of flooding protocols in WSNs. Besides, we compare CONE to the baseline protocol Trickle  [13] in order to address benefits brought by using a CDS. Note, we choose Trickle as the baseline because it is one of the most classic algorithms for propagating and maintaining code updates in WSNs and several IEEE 802.15.4 standards are based on Trickle, e.g., RPL [6].

In this work, we make the following contributions:

- We propose CONE, a connected dominating set-based flooding protocol for WSNs. CONE reduces the number of broadcasts by disallowing the nodes that are not in the CDS to rebroadcast packets during a flooding process.
- We implement CONE in Contiki OS [14] and carry out extensive experiments by simulation and in a real-world testbed, respectively.
- We evaluate the performance of CONE and compare it to the state-of-the-art in terms of end-to-end reliability and duty cycle in the simulator Cooja [15] and energy consumption in the testbed FlockLab [12].

In Section 2, we review several existing approaches for controlling a network’s topology. Also, we clarify the reasons why we choose a CDS-based approach in CONE. In Section 3, we describe the design of CONE. For this purpose, we detail the CDS construction and maintenance and then describe the portability of the CDS construction algorithm of CONE to other flooding protocols. In Section 4, we compare the performance of CONE and the baseline protocol [13] based on experiments in Cooja [15] and in FlockLab [12]. In Section 5, we summarize our work and provide a glimpse into future work.

## 2. Related Work

As previously mentioned, network flooding is widely applied in WSNs, but it is not energy-efficient enough. Increasing energy efficiency in WSNs is critical because of the limited power supply of nodes. Hence, providing reliable and energy-efficient flooding in WSNs is an important research topic in the research community [4,16,17]. Aiming at reliable flooding in low duty-cycle WSNs with unreliable wireless links, Cheng et al. propose a dynamic switching-based reliable flooding (DSRF) framework [16]. In DSRF, a flooding tree structure is dynamically adjusted based on the packet reception results to save energy and reduce delay, when encountering a transmission failure. Similarly, authors in [17] focus investigation on minimum-delay and energy-efficient flooding tree construction considering the duty-cycle operation and unreliable wireless links in WSNs. By formulating the problem as an undetermined-delay-constrained minimum spanning tree (UDC-MST) problem, they then design a distributed Minimum-Delay Energy-efficient flooding Tree (MDET) algorithm to construct an energy optimal tree with flooding delay bounding.

However, network flooding still suffers from broadcast storms which causes a large number of redundant messages and packet loss. Topology control aims to increase energy efficiency of network flooding and to reduce broadcast storms [8]. In this section, we review several methods of topology control. Topology control restricts the topology of a network with the goal to increase the network’s lifetime. The topology of a network is defined by its set of active nodes and active links between any two nodes. The optimal solutions to topology control methods are unfortunately NP-hard to find [8]. Hence, we also review approximation algorithms.

### 2.1. Power Control

One method of topology control is to save energy by reducing the transmission power of individual nodes. This method is referred to as “power control”. Power control reduces the number of active links, but it should also preserve connectivity and coverage of the network [11].

However, power control cannot always prevent the transmission of redundant packets in dense networks in regard to network flooding. Since in dense networks, nodes might be so close to each other that reducing the transmission power would not effectively reduce the number of active links. Hence, if nodes transmit packets simultaneously, they still might interfere the transmissions of each other, leading to packet loss. Therefore, power control cannot generally simplify a network’s topology in order to reduce broadcast storm [11].

### 2.2. Backbone Construction

Another method of topology control is that a network uses a so-called “backbone”. A backbone consists of nodes that perform data communication tasks and serve network nodes that are not part of the backbone. In the following sections, we describe a few methods of backbone construction.

#### 2.2.1. Independent Sets

**Figure 3:**
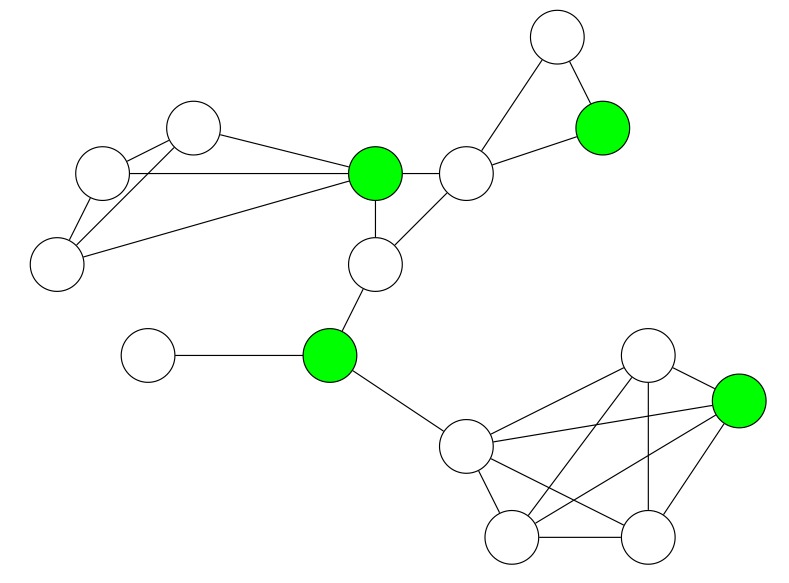
Maximal independent set. The nodes which form the MIS are colored in green.

An independent set (IS) contains nodes such that no pair of nodes within the set are adjacent [8]. If increasing the cardinality of an IS breaks the independence property, then the IS is regarded as a maximal independent set (MIS) (see Figure 3). If a node of a graph is not in the graph’s MIS, it is adjacent to a node of the graph’s MIS. Hence, every MIS is also a dominating set (DS) [11]. We discuss DSs further in Section 2.2.3. Since MISs are not connected, a network cannot use a MIS for propagating packets through a whole network like a CDS. However, a few CDS construction algorithms use a MIS in order to find a CDS. We discuss this further in Section 2.4.

#### 2.2.2. Spanning Trees

**Figure 4:**
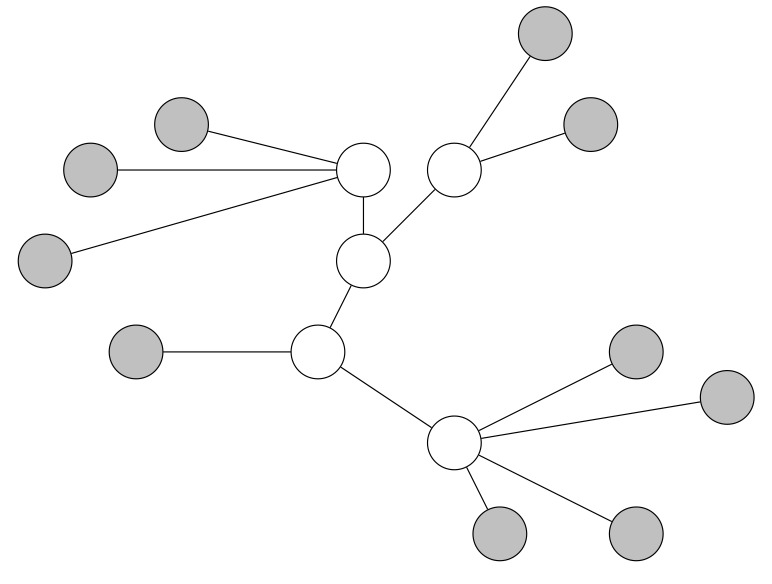
A spanning tree given by the white nodes. Leaf nodes are colored in grey.

Let a path be a totally ordered set that consists of several nodes. We regard one of these nodes as the beginning of the path and another node as the end of the path. A network can use a spanning tree (ST) to find an efficient path by reducing the number of edges in the graph. The path found could then be used to forward a packet to its destination. A ST is a connected subgraph of an initial network which contains all nodes (vertices) of the network (see Figure 4). A ST contains no circles (loops).

Many ST construction algorithms require that edges have non-negative weights. Some algorithms allow negative weights or do not require any weights at all. The weight of an edge represents the cost of transmitting a packet from one node to another node via this edge representing a communication link. The sum of weights of a path is regarded as the distance. Some ST algorithms also require a “root” which marks the beginning of the tree.

If the distance from the root to any other node of the ST is minimal, then the ST can be regarded as a shortest path tree (SPT). A similar structure is a minimum spanning tree (MST) which is a ST with the minimal sum of weights. However, SPTs and MSTs do not reduce the number of nodes, but the number of edges of all nodes. Because broadcasting still uses each edges of all nodes, flooding by SPTs and MSTs is not optimal [7].

A maximal leaf spanning tree (MLST) can be used to decrease the number of nodes. We regard nodes that are connected to only one node as a “leaf”. A MLST is a ST with the maximal number of leaves. In terms of network flooding, non-leaf nodes can be used to distribute a packet through the whole network. A MLST contains the minimal set of so-called “forwarders”. A forwarder is a node which forwards a received packet in the direction of its destination. The MLST problem is equivalent to the MCDS problem which also produces the minimal set of forwarder nodes. We discuss CDSs in the next section.

#### 2.2.3. Connected Dominating Sets

**Figure 5:**
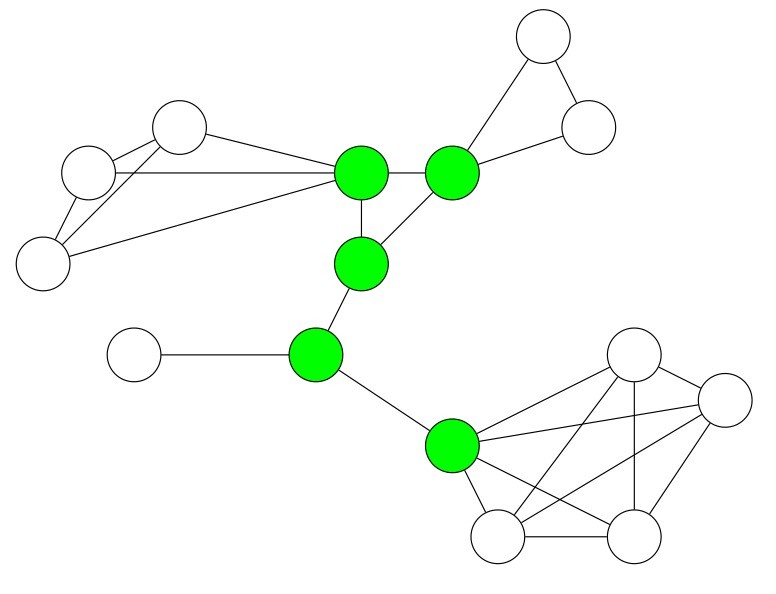
Connected dominating set. The dominators are colored in green.

A DS is a subgraph of a graph G that represents a network (see Figure 5). Each node of G is either in the DS or adjacent to a node of the DS. We refer to a node that is in the DS as a “dominator”. Accordingly, we refer to a node that is not in the DS as a “non-dominator”.

A DS is connected and, therefore, a CDS if there is a path from each dominator to each other dominator and this path includes only dominators. As mentioned in Section 1, a MCDS contains the minimal number of dominators that are required to cover the whole network. Finding a MCDS is referred to as the “MCDS problem” [11].

CDSs are usually used as a backbone [18]. As previously mentioned, many nodes in network flooding unnecessarily forward a packet which results in a broadcast storm. Using a CDS for network flooding can decrease broadcast storms by decreasing the number of forwarder nodes [11]. Furthermore, a node’s radio transceiver must be turned off as often and as long as possible in order to achieve a long lifetime of the node [19]. When a CDS is used, non-dominators can turn off their radio transceiver for a longer time period in order to save energy.

### 2.3. Clustering

**Figure 6:**
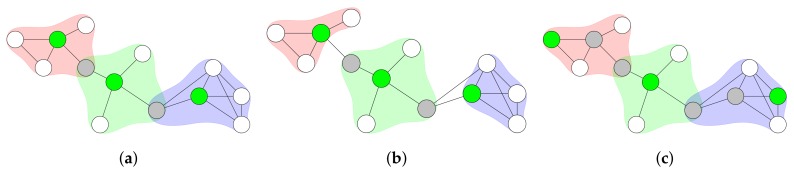
Network clustering. Colored areas around nodes represent clusters. Cluster heads are colored in green. Gateways or distributed gateways are colored in grey. (**a**) Clustering with overlapping clusters. (**b**) Clustering with non-overlapping clusters. (**c**) Clustering with distributed gateways.

A similar approach to CDS is called “clustering”. Clustering partitions a network into clusters. A cluster contains several nodes. One particular node in each cluster has the task to manage its cluster—it is called a “cluster head”. Figure 6 illustrates a network that uses clustering. If a node separates two cluster heads, it can assist in the communication between these two cluster heads. Therefore, the assisting node is referred to as “gateway”. Clusters might overlap (see Figure 6a), but non-overlapping clusters (see Figure 6b) are also possible. It can also be the case that two nodes separate a cluster head from the next cluster head. In this case, these two nodes form a so-called “distributed gateway” (see Figure 6c).

Two cluster heads can be neighbors, but it is often desirable that cluster heads are separated [8]. Therefore, the set of cluster heads ideally forms an IS. However, in terms of reducing the set of nodes, it is more useful to keep the number of cluster heads small. Hence, the set of cluster heads should not form a MIS, since this results in a larger number of cluster heads. Therefore, the cluster configuration without the MIS is more beneficial for reducing the set of nodes. The advantages of clustering are similar to the ones of using a backbone. In fact, CDSs are sometimes used in order to find the set of cluster heads and the set of gateways [8]. However, clustering puts more emphasis on enhancing scalability of higher-layer protocols and local resource allocation [8]. Hence, using clustering in network flooding does not provide any added value compared to using a CDS.

### 2.4. Tree Growing Algorithm

We choose a CDS-based approach, since exploiting a CDS provides us with the most benefits in regard to network flooding. Finding a MCDS is considered NP-hard [8]. Therefore, we need an approximation algorithm that keeps the set of forwarders as small as possible in order to reduce flooding redundancy effectively. There has been a lot of work done on finding good approximation algorithms for the MCDS problem [11]. The work of [11] provides us with three types of CDS construction algorithms: subtraction-based, MIS-based, and tree-based algorithms. Compared to subtraction-based and MIS-based algorithms, tree-based algorithms produce generally smaller CDSs and cause less message overhead [11]. Hence, we choose to implement a tree-based algorithm for CONE. We use the tree-based algorithm suggested by Guha & Khuller [20], referred to as “tree growing” algorithm. In the following, we describe two centralized variations of the tree growing algorithm.

#### 2.4.1. Greedy Variation

The tree growing algorithm iteratively adds nodes to the DS until it is connected. This algorithm colors nodes either in white, grey, or green in order to indicate the node’s state. White nodes are non-dominators. Grey nodes are also non-dominators, but they are adjacent to at least one dominator. Green nodes are dominators. Algorithm 1 shows the tree growing algorithm in pseudo-code.
**Algorithm 1** Greedy tree growing algorithm.1:initially color all nodes in white2:choose a node *m* with the largest number of white neighbors3:color node *m* in green and the white neighbors of node *m* in grey4:**while** white nodes exist **do**5:    choose a grey node *n* with the largest number of white neighbors6:    color node *n* in green and the white neighbors of node *n* in grey7:**end while**

**Figure 7:**
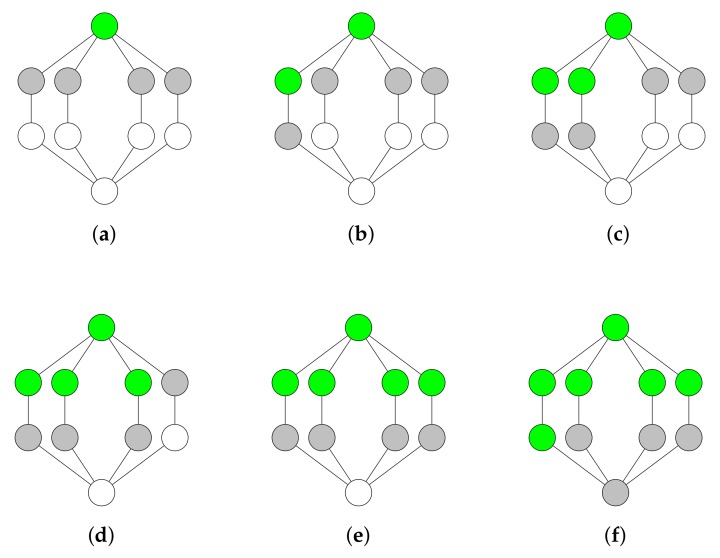
Tree growing algorithm. The dominators are colored in green. The non-dominators that are adjacent to at least one dominator are colored in grey. (**a**) The algorithm arbitrarily chooses a node with the largest number of white neighbors. The chosen node is colored in green and its neighbors are colored in grey. (**b**–**f**) The algorithm arbitrarily chooses a grey node with the largest number of white neighbors. The chosen node is colored in green and its white neighbors are colored in grey. The algorithm stops in (**f**) because each node is colored in either green or grey.

**Figure 8:**
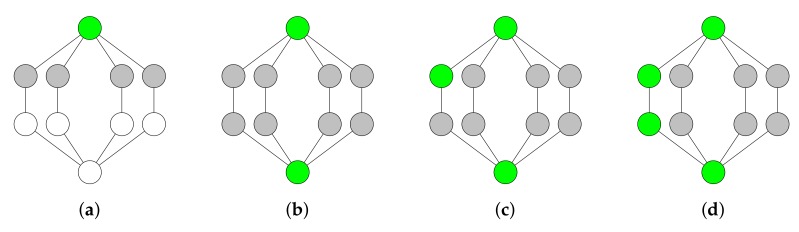
Enhanced tree growing algorithm. The dominators are colored in green. The non-dominators that are adjacent to at least one dominator are colored in grey. (**a**,**b**) The algorithm arbitrarily chooses a node with the largest number of white neighbors. The chosen node is colored in green and its neighbors are colored in grey. (**c**,**d**) The algorithm arbitrarily chooses a grey node with the largest number of green neighbors. The chosen node is colored in green. The algorithm stops in (**d**) because each node is colored in either green or grey and the set of green nodes is connected.

When all nodes are colored in green or grey, the algorithm has successfully constructed a CDS, represented by the set of green nodes. Figure 7 provides an example of the greedy tree growing algorithm. The advantage of this algorithm is that it keeps the set of dominators connected until a CDS has been constructed. The disadvantage of the algorithm is that choosing only grey nodes to become green nodes in Line 5 of Algorithm 1 results in a larger CDS for some topologies. Choosing both white and grey nodes to become green nodes would result in a smaller CDS in less rounds, as shown in Figure 8.

#### 2.4.2. Enhanced Variation

An enhanced variation of the tree growing algorithm iteratively adds grey and white nodes to the DS. Like the greedy tree growing algorithm, this algorithm colors node either in white, grey, or green in order to express the node’s state. White nodes are non-dominators. Grey nodes are also non-dominators, but are adjacent to at least one dominator. Green node are dominators. Algorithm 2 shows the enhanced tree growing algorithm in pseudo-code. When all nodes are colored in green or grey, the algorithm has successfully constructed a CDS. However, centralized CDS construction algorithms are usually not directly applicable to WSNs due to the absence of a central administration and possible large network size [21]. For this reason, we design and implement a fully distributed tree growing algorithm, which is based on the enhanced variation of the tree growing algorithm. We discuss the design of our algorithm in the next section.
**Algorithm 2** Enhanced tree growing algorithm.1:initially color all nodes in white2:**while** white nodes existing **do**3:    choose a grey or white node *n* with the largest number of white neighbors4:    color node *n* in green and the neighbors of node *n* in grey5:**end while**6:**while** dominating set is not connected **do**7:    choose a grey node *m* with the largest number of green neighbors8:    color node *m* in green9:**end while**

## 3. Design and Implementation of CONE

In this section, we describe the design of CONE. CONE exploits the Trickle algorithm [13] for network flooding. Trickle is a popular method for data dissemination in sensor networks [22]. It has been standardized as the mechanism that regulates the transmission of the control messages used to create the network graph in RPL [23]. Constrained Low-power and Lossy networks (LLNs) which includes WSNs represent the foundation for IoT that deploys RPL.

Optimizing Trickle has become a popular research topic [1,22,23,24]. Furthermore, simulations have shown that Trickle suffers from the broadcast storm problem, especially in dense network. For these reasons, our goal is to enhance Trickle by using a CDS.

We implement CONE in Contiki OS based on Tmote Sky sensor nodes. Contiki OS is an operating system for nodes which are used for the IoT [14]. We use the rime communication stack for network communication. Rime is a lightweight communication stack which has been designed for WSNs [25]. Furthermore, the implementation of Trickle in Contiki OS employs rime for communication as well.

To use a CDS, CONE has to construct a CDS in the first place. We describe the CDS construction in CONE in the following sections. In Section 3.1, we explain how CONE uses a CDS for the flooding process with Trickle. In Section 3.2, we detail the neighbor discover and information exchange in CONE. Furthermore, we have to consider node failures [26], since they can easily disconnect a CDS. In Section 3.3, we describe how CONE maintains a constructed CDS in case of node failures.

### 3.1. Connected Dominating Set Construction

Based on the centralized tree growing algorithm from [20], we design a distributed algorithm that works in WSNs. The algorithm constructs a DS and then adds nodes to the DS in order to connect the DS. For performing the DS construction, nodes have to know their 1-hop neighbors and their degrees. The degree of a node is defined as the number of a node’s known neighbors. In Section 3.2, we describe how nodes discover their neighbors and their degree. Then, we explain the DS construction in Section 3.2.1 and the DS connection in Section 3.2.2.

Our design does not assume any knowledge of the network topology due to a possibly large network size and random deployment of nodes in an area. For this reason, our algorithm uses only broadcasting for communication. Furthermore, CONE uses event timers and callback timers to randomly schedule transmission in order to reduce duty cycles and packet loss of nodes during CDS construction. An event timer sets a flag to true when it expires. If a callback timer expires, then it triggers a callback function.

### 3.2. Neighbor Discovery and Degree Exchange

As previously stated, nodes have to know their 1-hop neighbors and their degree to construct a DS. Algorithm 3 gives the pseudo-code of the neighbor discovery and degree exchange procedure. Nodes exchange their degree with their neighbors. For this purpose, a node periodically broadcast a degree_message which contains the sender’s degree. During the degree exchange, nodes also discover their neighbors. If a node discovers a new neighbor, then it updates its degree. Also, each node stores the node which has—up to that time—sent the highest degree in order to elect this node for becoming a dominator later.
**Algorithm 3** Neighbor discovery and degree exchange.1:receive degree_message2:**if** sender’s ID unknown **then**3:    mark sender’s ID as known4:    increase degree by 15:**end if**6:**if** received degree is greater than highest known degree **then**7:    consider sender as dominator8:    set highest known degree to sender’s degree9:**else if** received degree is equal to highest known degree **then**10:    **if** sender’s ID is lower than the one of current dominator **then**11:        consider sender as new dominator12:    **end if**13:**end if**

#### 3.2.1. Constructing a Dominating Set

In this step, nodes create a DS by electing a particular node to become dominator. Algorithm 4 gives the pseudo-code of the main process of a DS construction. Besides, on the sender’s side, the pseudo-code of the election process is shown in Algorithm 5. Nodes use an election_message which contains the identification (ID) of the sender’s dominator.
**Algorithm 4** Dominating set construction.1:**if** receive degree_message **then**2:    **while** degree is lower than threshold **and** timer for degree exchange has not expired yet **do**3:        broadcast degree_message4:        set timer for degree_message5:        wait until timer for degree_message expires6:    **end while**7:**end if**8:**if** receive election_message **then**9:    set callback timer for election10:    pass election_callback as a parameter11:    proceed with DS connection12:**end if**
**Algorithm 5** Neighbor election.1:receive election_message2:**if** dominator’s ID is equal to one of elected dominator **then**3:    **if** (sender’s ID is lower than my ID) **or** (sender’s ID is equal to my dominator’s) **then**4:        stop timer for election_message5:    **end if**6:**else if** elected dominator’s ID is equal to my ID **and** I am not a dominator **then**7:    mark me as dominator8:**end if**9:election_callback10:**if** timer for election has expired **then**11:    send election_message12:    set callback timer for election and pass election_callback as paramet13:**end if**

#### 3.2.2. Connecting the Dominating Set

If a node initializes the flooding process by broadcasting a packet, then not all nodes in the network are able to receive the packet. The resulting DS from the previous step is not necessarily connected. For instance, the green nodes in Figure 8b are not connected to each other via other nodes. To connect the DS, we have to choose additional nodes to become dominators.

The work from [20] uses a ST construction algorithm in order to connect the DS. However, this algorithm requires that each node knows all other nodes in the network. To keep the message overhead as low as possible, we came up with another idea for this work which requires less information. Our idea is that each node periodically broadcasts a token_message, in which way a token_value is updated. Every node in the network has a token_value which is the largest known ID of a dominator in the network. A node’s token_message contains the sender’s token_value. If all nodes in the network have the same token_value, then the DS_is_connected is true. Accordingly, if a received token_message differentiates from the receiver’s token_message, then it indicates that the flag DS_is_connected is false.

**Figure 9:**
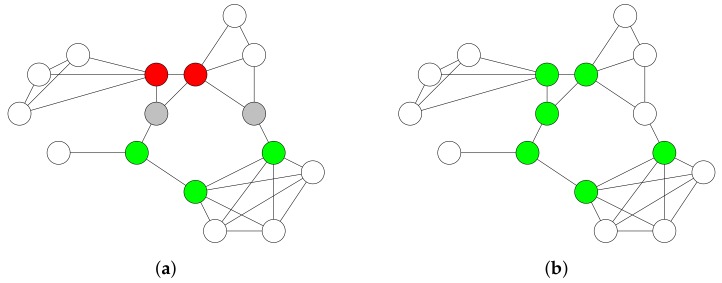
Dominating set being turned into a connected dominating set. The dominators are colored in green and red. The red dominators have a lower token value than the green dominators. White and grey nodes are non-dominators. Furthermore, the grey nodes separate the two disconnected sets of dominators. (**a**) The red dominators periodically receive the token with the largest value from the grey nodes, but do not update their token value because the grey nodes are not dominators. After receiving the token with the largest value from the grey nodes n times, the red dominators elect one of the grey nodes to become a dominator. When one of the grey nodes becomes a dominator, the red dominators accept the token with the largest value from it. (**b**) All dominators have the highest token value and thus are colored in green.

Algorithm 6 provides the pseudo-code of how nodes use their token_message in order to connect the DS. Each node periodically broadcasts a token_message. A token_message contains the sender’s token and a flag which is set if the sender is a dominator. Nodes update their token if they receive a greater token from a dominator. However, a node does not accept a token_message that was sent by a non-dominator. Figure 9 shows how nodes can use their token_message in order to connect their DS. Let the red nodes have a lower token_value than the green nodes. The red nodes will not accept the largest token_value, because there is no dominator that broadcasts the token_message to them. However, the grey nodes are in transmission range of the dominator with the largest token_value. Hence, the red nodes receive the higher token_value from the grey nodes. Furthermore, selecting one of the grey nodes results in the connection of the DS. To select a grey node, each red node increases its local counter by one if it receives a higher token_message from the grey nodes. If the counter reaches a predefined threshold, the node resets it to zero and selects one of the grey nodes. Also, the red nodes record the grey node’s ID which has the lowest ID in their neighborhood to increase the possibility that both red nodes select only one grey node.
**Algorithm 6** Token exchange.1:receive token_message2:**if** dominator’s ID equals the received token **then**3:    set ID of my dominator to 04:    stop timer for election_message5:**else if** (my dominator is the sender of the received token_message **and** sender is a dominator) **then**6:    set ID of my dominator to 07:    stop timer for election_message8:**end if**9:**if** value of my token is lower than the one of received token **then**10:    **if** sender is a dominator **then**11:        set value of my token to value of received token12:    **else**13:        **if** (ID of my dominator equals 0) **or** (ID of sender is equal to my dominator) **then**14:           set sender as my new dominator15:        **end if**16:        increase counter by 117:        **if** counter equals threshold **then**18:           set token counter to 019:           set counter to 020:           set timer for election_message21:        **else if** value of my token is greater than the one of received token **then**22:           set token counter to 023:        **end if**24:    **end if**25:**end if**26:periodically broadcast token_message27:**while** DS_is_connected is false **do**28:    set timer for broadcasting token_message29:    wait until (token counter is lower than threshold or I am a dominator)30:    wait until timer for broadcasting token_message has expired31:    **if** (DS_is_connected is false) **and** (token is not equal to 0) **then**32:        broadcast token_message33:        increase token counter by 134:    **end if**35:**end while**36:set ID of my dominator to 037:stop timer for election_message

### 3.3. Flooding Protocol

A node proceeds with the flooding process once an event timer for the CDS construction has expired. In flooding, an initiator is a node that initiates network flooding by initially broadcasting a message. During the flooding process, the initiator node periodically broadcasts a packet in order to trigger network flooding. For the flooding process, we use the Trickle algorithm [13].

**Figure 10:**
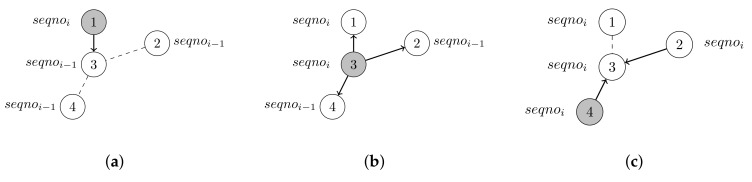
The Trickle protocol. The messages contain the nodes’ ID and sequence number $seqno_{x}$. The current sender is colored in grey. The dashed lines indicate that two nodes are in transmission range of each other. The thick arrows indicate a transmission from one node to another one. (**a**) Node 1 broadcasts information with $seqno_{i}$ to node 3. (**b**) Node 3 updates its local stored information after receiving the information from node 1 and rebroadcasts the message with $seqno_{i}$ to node 1, 2, and 4. (**c**) Node 2 and 4 update their locally stored information after receiving the message from node 3 and broadcast the message with $seqno_{i}$ to node 3.

Trickle uses polite gossip in order to distribute information through an entire network. Polite gossip means that if a node receives new or old information, then the node rebroadcasts the received information. Otherwise, the node does not broadcast the received information, because broadcasting the same information again is considered being aggressive. Figure 10 provides an example of how Trickle works. To determine whether information is old or new, packets in Trickle contain a sequence number seqno_x.

In case of CONE, only dominators rebroadcast information. As stated in Section 2.2.3, each node in the network that is not in the CDS is adjacent to at least one node of the CDS. Therefore, it is sufficient that only nodes in the CDS rebroadcast information in order to ensure that all nodes in the network receive the newest information. Hence, CONE can reduce redundant broadcasts in Trickle by using a CDS without impairing Trickle’s ability to keep information in a network up to date. Algorithm 7 shows how Trickle works in pseudo-code when it uses a CDS.
**Algorithm 7** Trickle using a CDS.1:receive Trickle_message2:**if** received sequence number is equal to my sequence number **then**3:    stop rebroadcasting4:**else if** received sequence number is lower than my sequence number **then**5:    **if** I am dominator **then**6:        broadcast information7:    **end if**8:**else**9:    set my sequence number to received sequence number10:    store the received information11:    **if** I am dominator **then**12:        set timer for broadcasting information13:    **end if**14:**end if**

### 3.4. CDS Maintenance

We designed CONE such that a network can maintain a constructed CDS. CONE can cover the following cases: (1) new node joins the network after CDS construction is done, (2) a dominator leaves the network because of node failure, and (3) a non-dominator loses connection to all dominators.

**Figure 11:**
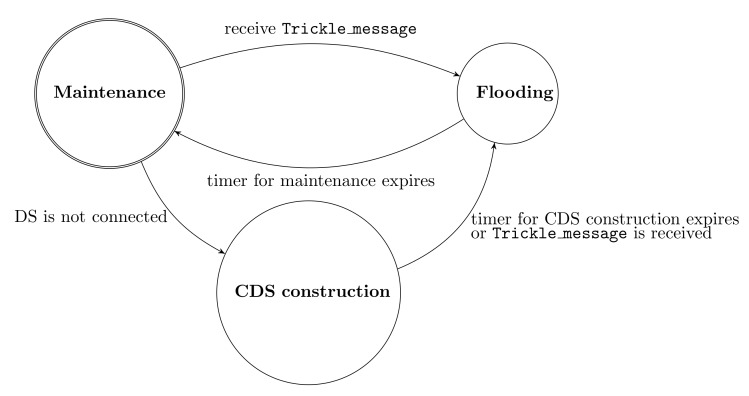
Flooding protocol states. The state transitions are caused by events which can occur when a function is called or when a variable is set. The state Maintenance is the starting state. The function “receives Trickle_message” handles messages received by Trickle.

Nodes use maintenance_messages and is_connected_messages in order to handle those three cases. The state chart in Figure 11 shows how CONE maintains a constructed CDS. When a node initializes CONE, then it broadcasts a maintenance_messages to ask its neighbors whether a CDS has been constructed. If a node receives a is_connected_messages, then this means that a CDS has been constructed and, therefore, the node does not need to initialize the CDS construction. Instead, it enters the flooding process. Furthermore, because the initiator broadcasts flooding packets in a known interval, other nodes in the network expect to receive flooding packets after a certain time. If this is not the case, then nodes broadcast a maintenance_messages to ask their neighbors whether the CDS is still intact. Nodes which receive a maintenance_messages reply with a is_connected_messages if the CDS is still intact. Algorithm 8 provides the pseudo-code for CDS maintenance.
**Algorithm 8** CDS maintenance.1:**while** True **do**2:    **if** DS_is_connected is false **then**3:        proceed with CDS construction4:        set timer for construction5:        wait until (DS_is_connected is true) or (timer for construction expires)6:    **else**7:        set timer for maintenance8:    **end if**9:    **if** (I’m not the initiator) **and** (DS_is_connected is true) **and** (timer for maintenance expired) **then**10:        set DS_is_connected to false11:        broadcast maintenance_message12:        set timer for maintenance13:        wait until (DS_is_connected is true) or (timer for maintenance expires)14:    **end if**15:**end while**16:**if** receive maintenance_message **then**17:    **if** DS_is_connected is true **then**18:        broadcast is_connected_message19:    **end if**20:**end if**21:**if** receive is_connected_message **then**22:    **if** (DS_is_connected is false) **and** (sender is not dominator) **then**23:        set sender as dominator24:        set callback timer for election25:        pass election_callback as parameter26:    **end if**27:**end if**28:**if** receive Trickle_message **then**29:    DS_is_connected is true30:    restart timer for broadcasting maintenance message31:    handle received message32:**end if**

## 4. Evaluation

In this section, we evaluate CONE based on results from simulations and from a real-world testbed. For this purpose, we compare the performance of CONE and our baseline protocol Trickle. Note, that we choose Trickle as the baseline because it is one of the most classic algorithms for propagating and maintaining code updates in WSNs and several IEEE 802.15.4 standards are based on Trickle, e.g., RPL [6]. However, CONE is flexible to adapt to other flooding protocols in Contiki. The difference between both protocols is that CONE additionally uses a CDS together with Trickle for network flooding. We use a Tmote Sky sensor platform in our simulations and testbed experiments, respectively. We first present our simulation results followed by our testbed results.

### 4.1. Simulations

We simulate CONE and Trickle by using the Cooja network simulator [15] in Contiki OS [14]. Cooja is often used in the WSN community for debugging and performance evaluation of WSN projects [27]. To see how CONE affects RDCs and packet loss, we compare the average RDC and average packet loss of all nodes in our simulations. Furthermore, we compare the largest measured RDC of nodes in CONE and in Trickle, respectively.

#### 4.1.1. Setup

For our simulations, we use a network that consists of 100 nodes in three scenarios: We simulate a network with various transmission ranges of 15 meters, 35 meters, and 45 meters, in order to evaluate the performance of CONE in different network densities. The nodes are arranged in a square in all scenarios. A larger transmission range results in a higher network density in our scenarios due to the larger number of neighbors of nodes. We expect that a higher network density leads to the occurrence of broadcast storms due to a larger number of simultaneous transmissions in a node’s neighborhood. Furthermore, a larger transmission range of nodes results in a smaller maximal hop distance of the network. The maximal hop distance of a network is the minimal number of hops needed to distribute packets from the initiator (source of information) to all other nodes in the network (i.e., covering the whole network). Each test runs for ten minutes. During those ten minutes, an initiator broadcasts one packet in every 15 seconds. CONE uses the first 90 seconds to construct a CDS.

#### 4.1.2. Results

**Figure 12:**
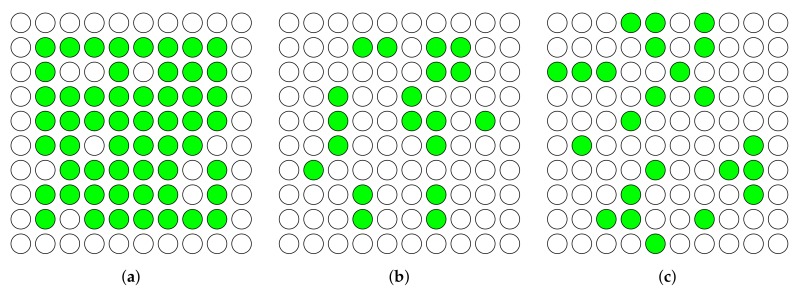
Constructed CDSs of three different network densities in simulations. The dominators are colored in green. Links between nodes are not illustrated due to their large number. A larger hop distance represents a smaller density of the network. Therefore, a network with a larger hop distance is required to have more dominators to cover the whole network. (**a**) Maximal hop distance: 9 hops. (**b**) Maximal hop distance: 5 hops. (**c**) Maximal hop distance: 3 hops.

Figure 12 illustrates the CDSs which have been constructed by CONE during our simulations. The green nodes represent the dominators. Due to the arrangement of nodes, the network contains several nodes that have similar sets of neighbors with the highest degree in their local neighborhood. Since nodes elect dominators with the lowest known ID, the intersection of two or more similar sets of neighbors does not collectively elect one dominator. This causes a larger set size for the constructed CDSs in all three scenarios.

As shown in the figure, with a lower network density (see Figure 12a), CONE requires 55 dominator nodes so as to cover the whole network. While with a higher network density (see Figure 12b,c), CONE needs much fewer dominators, i.e., 23 and 19, respectively. This indicates that CONE is able to decrease the number of nodes (dominators) that are responsible for broadcasting, in order to reduce broadcast redundancy and improve energy efficiency accordingly. The 9-hop scenario in Figure 12a has overall the largest number of dominators, since the network diameter is the largest one. Furthermore, the number of dominators of the 3-hop scenario in Figure 12c is larger than in the one of the 5-hop scenario in Figure 12b. The larger number of dominators in the 3-hop scenario occurs because we limit the degree of nodes in our implementation. Otherwise, nodes would spend too much time in order to exchange their degrees. The degree limit causes a larger number of nodes with the highest degree in their local neighborhood that also have similar sets of neighbors.

**Figure 13:**
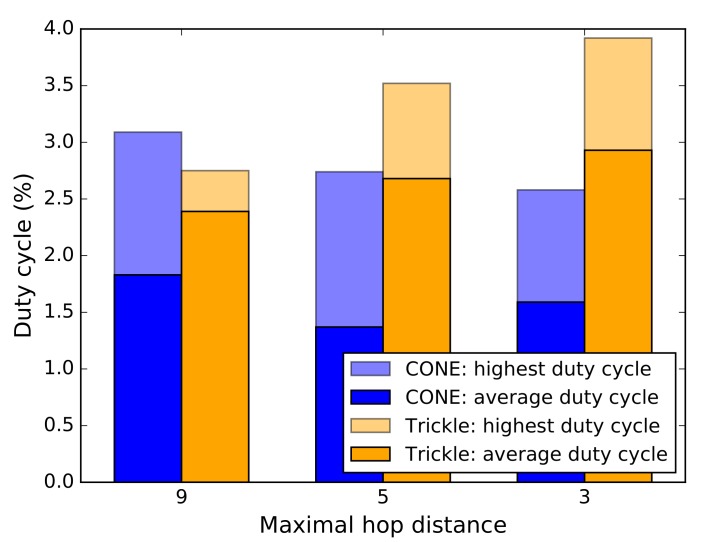
Comparison of average (radio) duty cycles in simulations.

**Figure 14:**
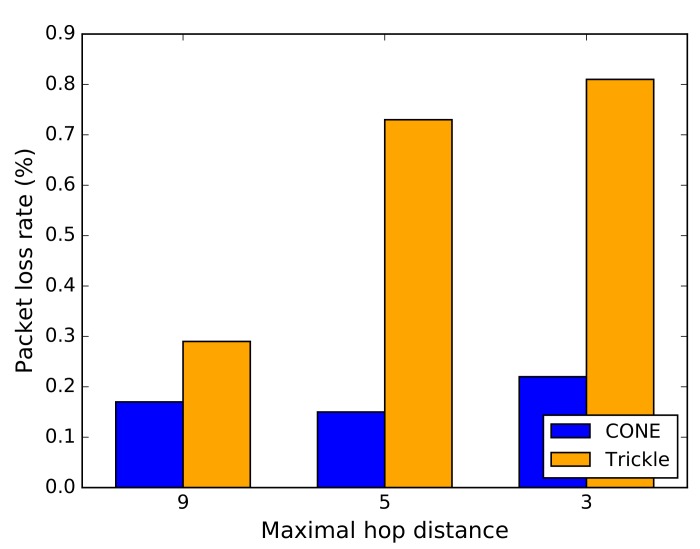
Comparison of average packet loss of nodes due to MAC contention.

CONE, in all scenarios, on average causes a smaller RDC and less packet loss compared to the baseline protocol (see Figure 13 and Figure 14). This means that CONE is able to effectively decrease packet loss by exploiting a CDS for network flooding. The nodes with the highest RDC in the scenarios from Figure 12b,c also have a smaller RDC, if we compare CONE and Trickle (see Figure 13). However, the node with the highest RDC in the scenario from Figure 12a has a slightly higher RDC for CONE.

However, higher RDCs occur only for dominators. Due to the lower number of nodes broadcasting packets in CONE, dominators receive packets more often compared to nodes in Trickle. This is shown in Figure 14. Additionally, dominators have to rebroadcast packets more often, which results in higher RDCs. However, the average RDC for CONE is still smaller compared to Trickle in the scenario of Figure 12a.

### 4.2. Experiments in the Testbed

**Figure 15:**
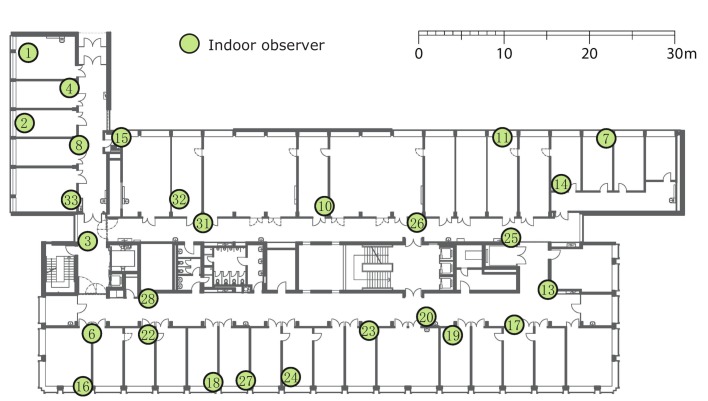
Deployment of sensor nodes in FlockLab.

To evaluate the energy consumption of CONE in a real-world scenario, we employ the FlockLab testbed introduced in [12]. The topology of sensor nodes in FlockLab is shown in Figure 15. FlockLab provides us with the average current consumption (in milliampere (mA)) of each individual node.

#### 4.2.1. Setup

**Figure 16:**
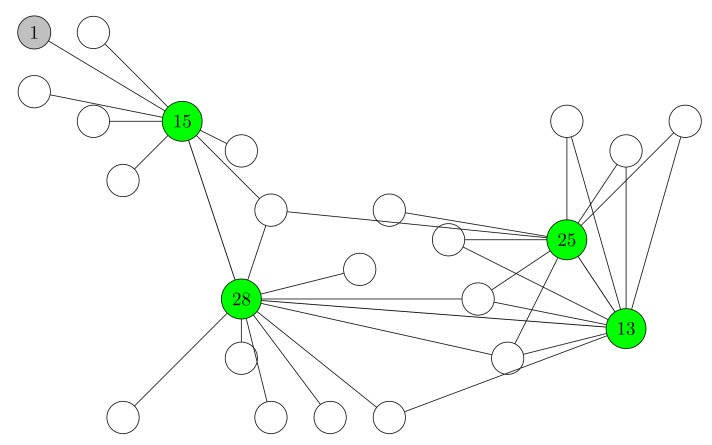
Constructed CDSs in the FlockLab experiments. The dominators are colored in green. The initiator is colored in grey. The edges indicate the active links between nodes. We only show active links between dominators and their neighboring nodes due to the large number of active links.

Our experiments in FlockLab use 22 nodes, since we observed that only 22 of the 25 nodes were available during all experiments. Each test runs for ten minutes. During these ten minutes, node 1 (see Figure 16)—as an initiator—broadcasts a packet every 15 seconds in order to flood the messages through the whole network. During the first 90 seconds, CONE uses the broadcasts to construct a CDS. After the first 90 seconds, CONE halts the CDS construction and starts the data dissemination by flooding. After ten minutes, we measure the average current consumption of all nodes.

#### 4.2.2. Results

As Figure 16 shows, in our experiment in FlockLab, CONE constructs a CDS with the green node 13, 15, 25, and 28 as dominators. Therefore, these nodes are many enough to cover the whole network, thus, continue to forward packets during the flooding process. Based on the connections between nodes, we can also see that a packet can flow from initiator 1 through dominators 15, 28, and 13 to dominator 25. Furthermore, every non-dominator is in transmission range of at least one dominator. This means that if a packet is forwarded by the dominators, then every other node in the network can receive that packet without retransmitting it. Thus, while in a flooding process, the dominators (in a CDS) receive packets from the initiator and forward the packets to all other nodes in the network. The non-dominators save a lot of energy during the flooding process by having their radio turned off for a longer time. Besides, this is sufficient to ensure that every node in the network is able to receive the packets from the initiator.

**Figure 17:**
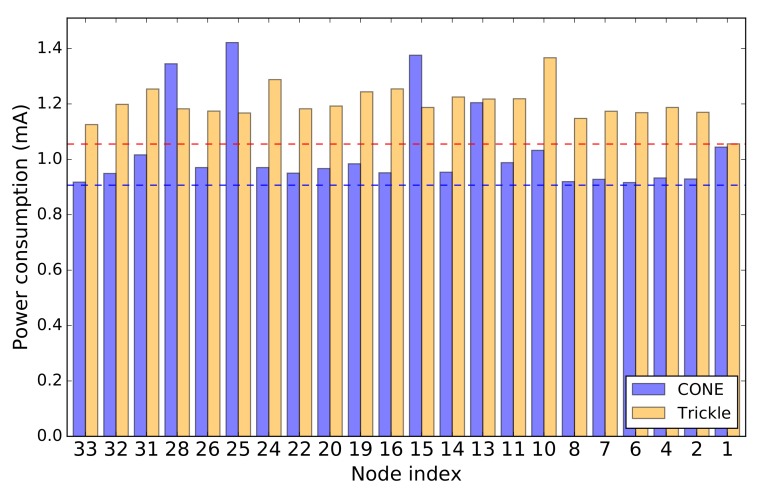
Comparison of energy consumption between CONE and the baseline protocol Trickle in FlockLab experiments. The dashed blue line shows the average energy consumption of all nodes in CONE while the dashed red line indicates the corresponding value in Trickle.

Figure 17 shows the energy consumption of the network in our FlockLab experiment after ten minutes. In CONE, the energy consumption of node 28, 25, and 15 is higher compared to the Trickle protocol. This could be expected due to the fact that these nodes are dominators and, therefore, have to forward packets so that the rest of the network can receive these packets. However, in CONE the energy consumption of all non-dominators is lower compared to Trickle. This results in an overall 15% lower average energy consumption for the network in CONE compared to Trickle. Because the radio transceiver of nodes consumes a significant amount of power when turned on, the non-dominators save energy by not retransmitting packets. With these results, we conclude that CONE decreases the average energy consumption in a network. However, the forwarding of packets during the flooding process is not distributed among all nodes in the network, but only among dominators. This means that dominators in CONE may have to forward packets more often compared to individual nodes in Trickle. Thus, CONE does not necessarily decrease the energy consumption of individual dominators.

## 5. Conclusions

The goal of this work was to improve the energy efficiency of WSN network flooding by exploiting a CDS on top of the flooding protocol. In this article, we presented the design and implementation of our CDS-based flooding protocol CONE. CONE constructs a CDS with only slight information of a network’s topology. Besides, we compared CONE with the baseline protocol Trickle, both in simulations and in a real-world testbed, in term of RDC, packet loss, and energy consumption. The results showed that CONE successfully decreases the number of lost packages for all nodes in the simulations. Testbed results demonstrated that CONE decreases the average energy consumption of a network during network flooding. However, CONE does not necessarily decrease energy consumption of dominator nodes.

As future work, we would like to improve CONE so that it can be used to construct a CDS while causing less overhead and at the same time resulting in a smaller-sized CDS. Additionally, we are interested in applying CONE on the top of concurrent transmission protocols, e.g., Glossy [28] and DeCoT [29]. Then, we are curious to compare CONE to the machine learning-based flooding protocol, e.g., LiM [30], which floods packets based on a superset of CDS in the network, and to further evaluate the performance of both.
